# Reduced Synapse and Axon Numbers in the Prefrontal Cortex of Rats Subjected to a Chronic Stress Model for Depression

**DOI:** 10.3389/fncel.2018.00024

**Published:** 2018-01-30

**Authors:** Dávid Csabai, Ove Wiborg, Boldizsár Czéh

**Affiliations:** ^1^MTA – PTE, Neurobiology of Stress Research Group, Szentágothai Research Centre, University of Pécs, Pécs, Hungary; ^2^Department of Clinical Medicine, Aarhus University, Aarhus, Denmark; ^3^Department of Health Science and Technology, Aalborg University, Aalborg, Denmark; ^4^Institute of Laboratory Medicine, Medical School, University of Pécs, Pécs, Hungary

**Keywords:** chronic mild stress, depressive disorder, electron microscope, infralimbic cortex, neuroplasticity, synaptic density, synaptic plasticity

## Abstract

Stressful experiences can induce structural changes in neurons of the limbic system. These cellular changes contribute to the development of stress-induced psychopathologies like depressive disorders. In the prefrontal cortex of chronically stressed animals, reduced dendritic length and spine loss have been reported. This loss of dendritic material should consequently result in synapse loss as well, because of the reduced dendritic surface. But so far, no one studied synapse numbers in the prefrontal cortex of chronically stressed animals. Here, we examined synaptic contacts in rats subjected to an animal model for depression, where animals are exposed to a chronic stress protocol. Our hypothesis was that long term stress should reduce the number of axo-spinous synapses in the medial prefrontal cortex. Adult male rats were exposed to daily stress for 9 weeks and afterward we did a post mortem quantitative electron microscopic analysis to quantify the number and morphology of synapses in the infralimbic cortex. We analyzed asymmetric (Type I) and symmetric (Type II) synapses in all cortical layers in control and stressed rats. We also quantified axon numbers and measured the volume of the infralimbic cortex. In our systematic unbiased analysis, we examined 21,000 axon terminals in total. We found the following numbers in the infralimbic cortex of control rats: 1.15 × 10^9^ asymmetric synapses, 1.06 × 10^8^ symmetric synapses and 1.00 × 10^8^ myelinated axons. The density of asymmetric synapses was 5.5/μm^3^ and the density of symmetric synapses was 0.5/μm^3^. Average synapse membrane length was 207 nm and the average axon terminal membrane length was 489 nm. Stress reduced the number of synapses and myelinated axons in the deeper cortical layers, while synapse membrane lengths were increased. These stress-induced ultrastructural changes indicate that neurons of the infralimbic cortex have reduced cortical network connectivity. Such reduced network connectivity is likely to form the anatomical basis for the impaired functioning of this brain area. Indeed, impaired functioning of the prefrontal cortex, such as cognitive deficits are common in stressed individuals as well as in depressed patients.

## Introduction

The medial prefrontal cortex (mPFC) coordinates several higher-order cognitive functions and controls the neuroendocrine, autonomic and behavioral response to stress (Dalley et al., 2004; McKlveen et al., 2013, 2015; Riga et al., 2014). Stress affects the functioning and cellular integrity of mPFC neurons (Holmes and Wellman, 2009; McEwen and Morrison, 2013; Lucassen et al., 2014; Arnsten, 2015). The best described stressed-induced cellular changes are the reduction of apical dendritic length and complexity of layer II–III pyramidal neurons, which is typically accompanied by reduced spine density (Cook and Wellman, 2004; Radley et al., 2004, 2006; Liston et al., 2006; Liu and Aghajanian, 2008). Furthermore, it has been shown that NMDA receptor activation is crucial for the stress-induced dendritic atrophy (Martin and Wellman, 2011). Finally, these stress-induced cellular changes in the mPFC correlate with executive dysfunctions (Holmes and Wellman, 2009).

The infralimbic (IL) cortex is the most ventral part of the mPFC and it is known to contribute to the coordination of the chronic stress response (Flak et al., 2012). Neurons of the IL cortex are particularly sensitive to chronic stressors (Hinwood et al., 2011) and it is well documented that stress disrupts the functioning of this cortical area (Izquierdo et al., 2006; Wilber et al., 2011; Koot et al., 2014; Moench et al., 2015). Stress-induced dendritic atrophy of layer III pyramidal neurons has been repeatedly demonstrated in the IL cortex together with the stress-induced loss of dendritic spines (Radley et al., 2004; Izquierdo et al., 2006; Perez-Cruz et al., 2007, 2009; Dias-Ferreira et al., 2009; Goldwater et al., 2009; Shansky et al., 2009). Because of the obvious regressive structural changes of the pyramidal neurons, synapse loss is also taken for granted in the mPFC of stressed rats. For example, Radley et al. (2006) estimated that repeated stress should produce a 33% reduction in the total number of axo-spinous synapses on the apical dendrites of pyramidal neurons. However, so far there is no experimental evidence confirming such expectations on fronto-cortical synapse loss. There are very few studies in the literature that focused directly on stress-induced synaptic changes in the mPFC. A recent report, which investigated the influence of acute stress on excitatory synapses, actually found *increased* synapse numbers within an hour after stress (Nava et al., 2014). Yet another study, which investigated inhibitory neurotransmission in the IL of chronically stressed rats, also found an *increased* number of inhibitory synaptic contacts onto glutamatergic cells (McKlveen et al., 2016). A developmental study documented synaptic rearrangements (higher spine synapses and fewer dendritic shaft synapses) in the anterior cingulate cortex of *Octodon degus* which were maternally deprived and reared in social isolation (Helmeke et al., 2001). The same experimental paradigm resulted in significantly *higher* synaptic densities in layer II of the IL cortex (Ovtscharoff and Braun, 2001).

Numerous scientists suggested that synaptic changes in the prefrontal cortex are key factors contributing to the pathophysiology of depressive disorders (Moghaddam, 2002; Popoli et al., 2011; Duman and Aghajanian, 2012; Duman, 2014; Thompson et al., 2015; Duman et al., 2016). Indeed, a recent post-mortem electron microscopic study demonstrated lower number of synapses in the dorsolateral prefrontal cortex of patients with major depressive disorder (Kang et al., 2012). Animal models based on chronic-stress paradigms are valuable tools to investigate the neurobiology of depressive disorders (Czéh et al., 2016). Here, we used the chronic mild stress (CMS) model, which is one of the best validated animal models for depression (Willner et al., 1992; Willner, 1997, 2005, 2016a,b; Wiborg, 2013) and studied the number and morphology of asymmetric (Type I, excitatory) synapses and symmetric (Type II, inhibitory) synapses in the ventral mPFC of rats. We did a detailed, systematic, quantitative electron microscopic (EM) analysis to examine the effect of long-term stress on synaptic numbers and morphology in all cortical layers of the IL cortex. Our hypothesis was that stress should reduce the number of axo-spinous excitatory synapses and probably also alter synaptic morphology. We also analyzed the number of myelinated axons, because recent studies documented stress-induced white matter changes (Miyata et al., 2016; Gao et al., 2017; Xiao et al., 2018).

## Materials and Methods

### Ethics Statement

Animal procedures and experiments were carried out in accordance with Aarhus University (Aarhus, Denmark) guidelines, Danish and European legislation regarding laboratory animals and approved by Danish National Committee for Ethics in Animal Experimentation (2008/561-447).

### Animals

Adult male Wistar rats (5–6 weeks old, with a body weight of about 120 g) were obtained from Taconic (Denmark). Eight rats were used in the present study: *n* = 4 controls and *n* = 4 chronically stressed. These eight animals were selected from a much larger cohort of animals that were all subjected to the same experimental procedures. We selected the four stressed animals based on their pronounced anhedonic behaviors (the behavioral phenotyping of the animals is described below). All animals were singly housed, except when grouping was applied as a stress parameter. We used single housing because we wanted to measure the sucrose intake of each individual rat. Food and water was available *ad libitum* except when food and/or water deprivation was applied as a stress parameter. The standard 12-h light/dark cycle was only changed in course of the stress regime. Results from these animals have been presented earlier in Csabai et al. (2017).

### Behavioral Phenotyping

Animals were behaviorally phenotyped with the use of the sucrose consumption test to detect their anhedonic behavior in response to stress. Stress-induced reduction of sucrose consumption indicates depressive-like anhedonic behavior. Before the real testing started all rats were trained to consume a palatable sucrose solution (1.5%). This training lasted for 5 weeks, with testing twice a week during the first 2 weeks and one test per week during the last 3 weeks. Animals were food and water deprived 14 h before the test. During the test the rats had free access to a bottle with 1.5% sucrose solution for 1 h. During the entire stress period, the sucrose consumption test was performed once a week. Baseline sucrose consumption was defined as the mean sucrose consumption during three sucrose tests conducted before starting the stress procedures.

### Chronic Mild Stress (CMS) Procedures

The CMS is one of the best described and most thoroughly validated animal models for depression (Willner et al., 1992; Willner, 1997, 2005, 2016a,b; Wiborg, 2013). The CMS procedure of our laboratory has been described in detail in our previous publications (see e.g., Henningsen et al., 2009, 2012; Wiborg, 2013; Csabai et al., 2017). Briefly, rats were divided into two matched groups on the basis of their baseline sucrose intake, and housed in separate rooms. One group of rats was exposed to 9 weeks of mild stressors. A second group of rats (controls) was left undisturbed. The schedule of the CMS was: a period of intermittent illumination, stroboscopic light, grouping, food or water deprivation; two periods of soiled cage and no stress; and three periods of 45° cage tilting. During grouping, rats were housed in pairs with different partners, with the individual rat alternately being a resident or an intruder. All the stressors lasted from 10 to 14 h. Based on the sucrose consumption, the hedonic state of the animals was evaluated and stressed rats were then further divided into stress sensitive rats (anhedonic animals) and stress-resilient rats (Henningsen et al., 2012). Anhedonic animals are the ones that reduce their sucrose solution intake by more than 30% in response to stress.

### Perfusion and Brain Tissue Preparation for the Electron Microscopic Analysis

We prepared the brain tissues for the ultrastructural analysis as described in detail before (Csabai et al., 2017). After an overdose of sodium pentobarbital (200 mg/ml dissolved in 10% ethanol) animals were transcardially perfused with ice cold 0.9% physiological saline followed by 4% paraformaldehyde containing 0.2% glutaraldehyde in 0.1 M phosphate buffer (pH 7.4). The brains were removed and postfixed overnight in the same solution at 4°C, but without glutaraldehyde. Serial, 80 μm thick, coronal sections were cut using a Vibratome (Leica VT1200 S) throughout the entire prefrontal cortex from Bregma level 4.70–2.20 (Paxinos and Watson, 1998). Two sections that included the IL cortex were selected from these section series and osmicated (1% OsO_4_ in PB for 30 min) and then, dehydrated in graded ethanol (the 70% ethanol contained 1% uranyl acetate). After complete dehydration in ascending ethanol series, the sections were immersed in propylene-oxide and then, into a mixture of propylene-oxide and Durcupan resin. Finally, they were flat-embedded in Durcupan resin (Fluka-Sigma–Aldrich, Hungary). After polymerization at 56°C for 48 h, the sections were viewed under a light microscope, and areas of interest were chosen for re-embedding and electron microscopic sectioning. To select the appropriate region of the IL cortex for ultrathin sectioning, semithin (500 nm) sections were stained with toluidine blue. The ultrathin (60 nm) sections were cut with a Leica Ultracut UCT microtome and collected on Formvar-coated single slot copper grids, stained with uranyl-acetate and lead citrate.

### Quantitative Analysis of the Synapses

**FIGURE 1:**
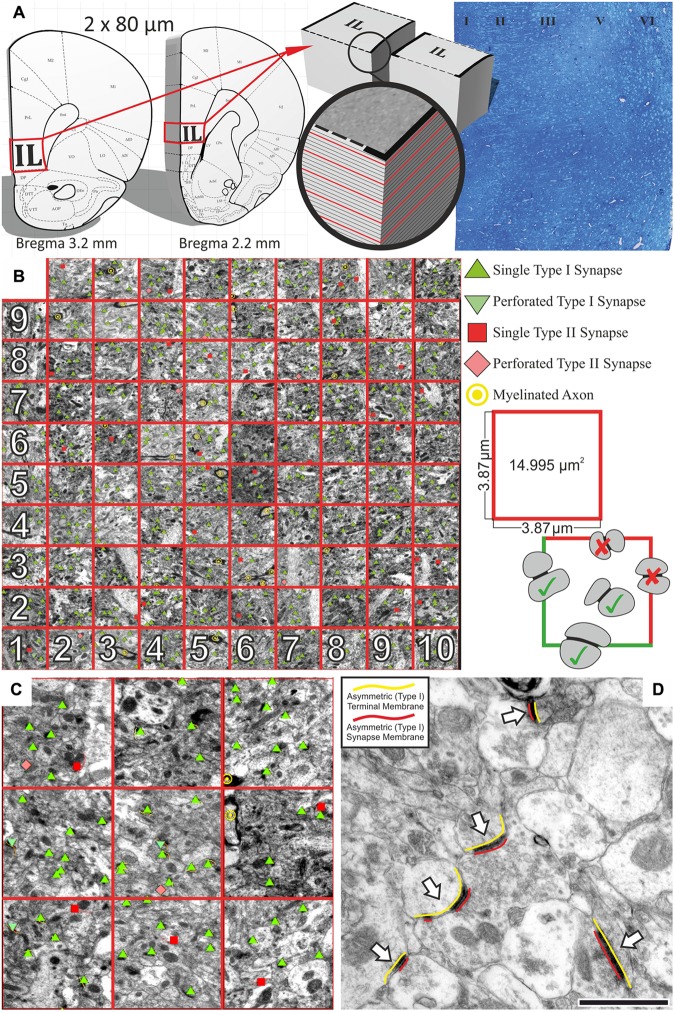
Systematic quantitative analysis of synapses in the IL cortex. **(A)** First, we selected two 80 μm thick coronal sections between Bregma levels of 3.20–2.20 mm. Then, we cut out the IL cortex from these sections and re-embedded them in durcupan resin and processed them further for ultra-sectioning. Semi-thin sections were cut from the durcupan embedded blocks and stained with toluidine blue dye to determine the exact area for ultra-sectioning. The six cortical layers were clearly identifiable in the toluidine blue stained sections. After orientation in the blocks, serial 60 nm ultrathin sections were cut and every fifth sections were collected on single slot copper grids (red sections). From each animal, we examined 10 ultrathin sections with the electron microscope. **(B)** In every ultrathin section, in each cortical layer, we made at least 10 non-overlapping photomicrographs using a sampling line with a random starting point. We analyzed 65–80 photomicrographs in each cortical layer of each animal. Synapses were counted and measured in these pictures with 40,000× magnification. All synapses were counted within an unbiased counting frame which had an area of ∼15 μm^2^ (3.87 μm × 3.87 μm). Synaptic profiles touching the exclusion (red) lines were not counted. Perforated synapses were also quantified, but these were few (5–10%) and their presence showed high individual variations. Therefore, we did not present data on them. **(C)** The quantitative analysis was done under the visual control of a single experimenter who used the Neurolucida software for this work. We quantified and measured asymmetric (Type I) and symmetric (Type II) synapses separately. Symmetric synapses were identified based on the morphology of the postsynaptic density and the shape of synaptic vesicles. **(D)** We measured synaptic junction lengths and the associated axon terminal membrane length (**Figures 2E,F**). Scale bar: 500 nm.

**FIGURE 2:**
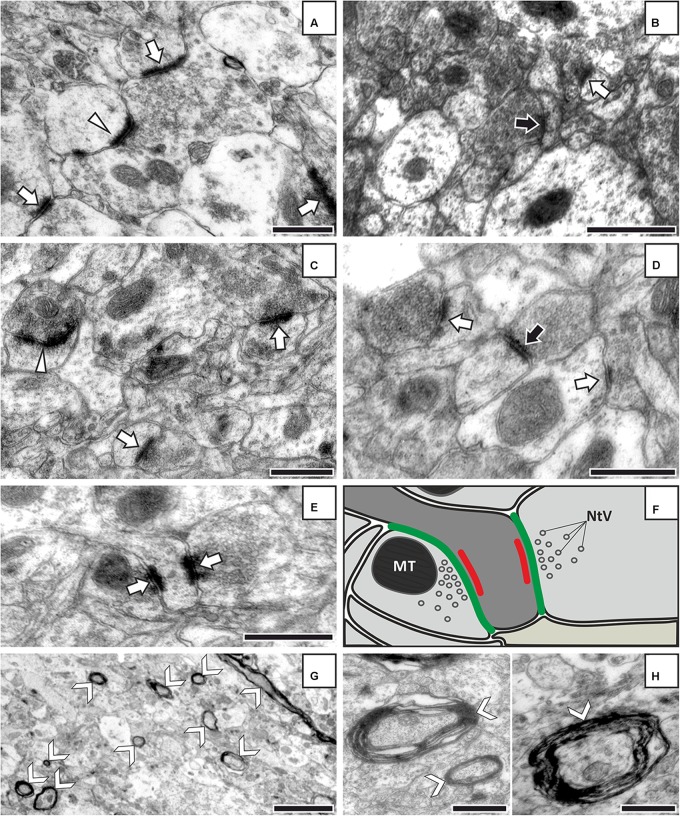
Representative EM images from control **(A,B)** and stressed rats **(C,D)** showing different types of synapses in cortical layers II–III. **(E)** Two axon terminals make synaptic contacts with one dendritic spine. **(F)** Simplified drawing of the EM image shown in **(E)**. The drawing illustrates how we measured synapse and axon terminal membrane lengths. Green lines represent the axon-terminal membrane and red lines indicate the synaptic membrane. **(G)** Myelinated axons are clearly visible in this low magnification EM image. Magnification: 5,000×, scale bar: 2 μm. **(H)** Higher resolution images of myelinated axons displaying the exact shape and electron density of the myelin sheats. White arrows point to asymmetric (Type I) synapses and black arrows indicate symmetric (Type II) synapses. The white arrowhead (on **A**) points to a perforated asymmetric synapse. Arrow nocks **(G,H)** show myelinated axons. MT, mitochondrium; NtV, neurotransmitter vesicles. The magnification was 40,000× for all images except of **(G)**. Scale bars: 500 nm on all images except of **(G)**.

All samples were coded before the quantification. The investigator analyzing the data was blind to the identity of the animals throughout the entire data analysis. We used the size-frequency method to quantify the numerical density of synapses per cortical unit volume (μm^3^). We applied this method because DeFelipe et al. (1999) demonstrated that this method is a solid method to quantify synapses in the neocortex and in fact it is more efficient and easier to apply than the disector method. First, we did a systematic random sampling protocol when cutting the brains and making the EM micrographs (**Figure 1**). We selected two 80 μm thick coronal sections between Bregma levels of 3.20–2.20 (Paxinos and Watson, 1998). These two sections were flat-embedded in Durcupan resin and then, they were cut further serially into ultrathin (60 nm) sections. From each of these two series of ultrathin sections we selected minimum 5–5 evenly distributed sample sections from each animal (**Figure 1A**). The selected ultrathin sections had approximately 5–6 μm distances between them. From each animal, we examined 10 ultrathin sections with a JEOL 1200 EX-II electron microscope. In every section, in each cortical layer, we made at least 10 non-overlapping EM photomicrographs using a sampling line with a random starting point. We analyzed 65–80 EM images in each cortical layer from each animal. In other words, we examined 400–500 EM pictures in the entire IL cortex of each animal (**Table 1**). The six cortical layers of the IL cortex were identified based on the description given by Gabbott et al. (1997). Synapses were counted and measured with 40,000× magnification. Synapses were counted within an unbiased counting frame (Gundersen, 1977), which covered ∼15 μm^2^ (3.87 μm × 3.87 μm) cortical area (**Figure 1B**). Synaptic profiles touching the exclusion lines were not counted. The quantitative analysis of the EM images was done under the visual control of a single experimenter (DC) who used the Neurolucida software (Version 11.08.2, MicroBrightField, Williston, VT, United States) for this work. We quantified and measured asymmetric (Type I) and symmetric (Type II) synapses separately (**Figure 2**). Symmetric synapses were identified based on the morphology of the postsynaptic density and the shape of synaptic vesicles (**Figure 2**). Furthermore, we measured synaptic junction length (the length of paired membrane densities at each junction) and the associated axon terminal membrane length (**Figures 1C**, **2E,F**). We also counted the number of myelinated axons (**Figures 2G,H**). The results of our quantitative data are reported here as synaptic densities as well as total synapse numbers. Similarly, the quantitative data on the number of myelinated axons are reported here as myelinated axon densities as well as total number of myelinated axons. Total synapse and axon numbers were calculated by multiplying the densities with the volume of the IL cortex (volume × density = total number).

**Table 1:** Results of quantitative EM analysis: Individual values.

	Control 1	Control 2	Control 3	Control 4	Stress 1	Stress 2	Stress 3	Stress 4
Number of analyzed sample areas^∗^	493	475	487	404	415	450	402	405
Number of analyzed axon terminals	4,600	2,267	2,632	2,256	2,312	2,397	1,867	2,321
Density of all synapses (n/μm^3^)	6.60	5.29	5.99	6.00	6.16	5.90	5.19	6.37
Total number of asymmetric synapses	1.22 × 10^9^	1.03 × 10^9^	1.19 × 10^9^	1.16 × 10^9^	1.05 × 10^9^	1.06 × 10^9^	0.92 × 10^9^	0.92 × 10^9^
Density of asymmetric synapses (n/μm^3^)	5.94	4.89	5.56	5.47	5.65	5.54	4.73	5.84
Total number of symmetric synapses	1.36 × 10^8^	0.84 × 10^8^	0.93 × 10^8^	1.12 × 10^8^	0.94 × 10^8^	0.68 × 10^8^	0.89 × 10^8^	0.83 × 10^8^
Density of symmetric synapses (n/μm^3^)	0.66	0.40	0.43	0.53	0.51	0.36	0.46	0.53
Terminal membrane length (nm)	469.4 ± 7.2	470.4 ± 15.5	500.5 ± 15.8	514.4 ± 15.9	450.2 ± 9.0	486.9 ± 16.3	480.2 ± 14.9	511.1 ± 14.0
Synapse membrane length (nm)	211.8 ± 2.4	205.0 ± 5.65	204.1 ± 5.2	207.7 ± 4.5	213.7 ± 3.4	203.4 ± 5.0	219.9 ± 6.0	211.9 ± 4.5
Total number of myelinated axons	0.70 × 10^8^	1.09 × 10^8^	1.19 × 10^8^	0.99 × 10^8^	0.82 × 10^8^	0.69 × 10^8^	0.64 × 10^8^	0.59 × 10^8^
Density of myelinated axons (n/μm^3^)	0.34	0.52	0.54	0.48	0.47	0.35	0.32	0.36


*Data are expressed as mean ± SEM. The total synapse numbers were calculated by multiplying synapse densities with the volume of the IL cortex. ^∗^Each sample area was 14.995 μm^*2*^.*

In our EM analysis, we also looked for evidences of neuronal degeneration or cell death in the samples from the stressed rats. Apoptotic cell death is indicated by the condensation of chromatin (electron-dense, black structure along the nuclear membrane) and fragmentation of the cell nucleus. Apoptotic cell bodies are densely packed with cellular organelles and nuclear fragments that are engulfed by phagocytosis of surrounding cells. The typical signs of neurodegeneration include swollen mitochondria with disorganized structure and disrupted cristae, or the presence of autophagic vacuole and electron dense degenerating (shrunken) neurites. Such cellular malformations are obvious and easy to recognize on the EM pictures.

### Volume Measurement

We measured the volume of the IL cortex based on Cavalieri’s principle (Gundersen et al., 1988). From each animal, we collected a series of 80 μm thick coronal sections covering the entire IL cortex starting from 3.5 mm to 2.0 mm relative to Bregma (Paxinos and Watson, 1998). We used every third serial section for the volume measurement, i.e., 5–6 sections/animal. These sections were Nissl stained and analyzed with a Nikon Eclipse Ti-U microscope, using a 4× objective. In each section, we measured the cross-sectional areas of cortical layer I, II, and III–VI within the IL. The borders between the different cortical layers were identified based on the description given by Gabbott et al. (1997). We also measured the cross-sectional area of the entire IL cortex. Cortical volumes were calculated by multiplying the cross-sectional areas with the thickness of the sections.

### Statistical Analysis

Results are presented here as the mean ± SEM. Since we expected that stress should reduce the number of synapses and the volume of the IL cortex, thus, these data were compared with a one-tailed unpaired Student *t*-test. Group values of axon numbers were compared with two-tailed unpaired Student *t*-test. The laminar distributions of the synaptic parameters were analyzed with a two-way ANOVA (stress × cortical layer) followed by Sidak *post hoc* test. Results of the sucrose consumption behavioral test were analyzed with a two-way ANOVA (stress × time) followed by Bonferroni’s *post hoc* test. We used parametric tests for our data analysis because of the following reasons: (1) Other studies quantifying stress-induced synaptic changes also use parametric tests (e.g., Hajszan et al., 2009; Nava et al., 2014; Baka et al., 2017); (2) We had only four animals/group, but in each animal we did 400–500 measurements and the results of these measurements showed normal distribution; (3) Non-parametric tests have very low statistical power when the number of individuals is low.

## Results

### Rats Subjected to Chronic Mild Stress Developed Depressive-Like Anhedonic Behavior

**FIGURE 3:**
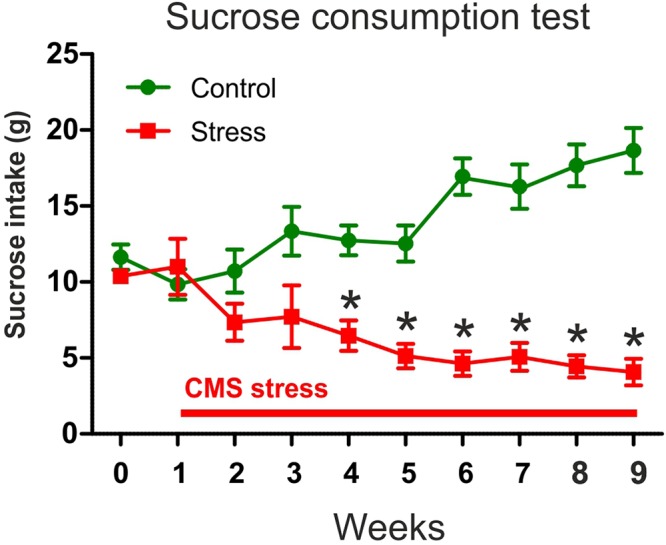
In response to chronic stress, the animals gradually developed depressive-like anhedonic behavior. The graph shows the sucrose consumption of the animals that were later used for the EM analysis. Baseline sucrose consumption was defined as the mean sucrose consumption during three sucrose tests conducted before stress initiation. Control rats gradually increased their sucrose intake indicating a healthy hedonic behavior. Stressed animals progressively reduced their sucrose intake which indicates anhedonic behavior. Two-way ANOVA (stress × time) revealed significant main effect of stress (*P* < 0.0001) and significant interaction with time (*P* < 0.0001). Bonferroni’s *post hoc* comparisons demonstrated that stressed rats consumed significantly less sucrose on week 4–9 compared to controls (^∗^*P* < 0.05).

We used the sucrose consumption test to assess the hedonic-anhedonic behavior of the animals. Nine weeks of CMS significantly decreased sucrose intake. In **Figure 3**, we present the sucrose intake data of the rats that were used here for the subsequent quantitative EM analysis. Two-way ANOVA (stress × time) revealed significant main effect of stress [*F*_(1,9)_ = 51.81, *P* < 0.0001] and significant interaction with time [*F*_(1,9)_ = 24.17, *P* < 0.0001]. Bonferroni’s *post hoc* comparisons demonstrated that the stressed rats consumed significantly less sucrose from the stress week 4 onward compared to control rats (**Figure 3**). The results of the statistical comparisons were the following: week 4 (*t* = 3.53, *P* < 0.01), week 5 (*t* = 4.18, *P* < 0.001), week 6 (*t* = 6.94, *P* < 0.001), week 7 (*t* = 5.71, *P* < 0.001), week 8 (*t* = 7.46, *P* < 0.001), and week 9 (*t* = 8.22, *P* < 0.001). These data indicate that CMS stress had significant consequences on the hedonic behavior of the animals and that the stressed rats gradually developed an anhedonic depressive-like behavioral phenotype.

### Quantitative EM Analysis

Examples of representative electron micrographs from the present experiment are shown in **Figure 2**. We analyzed 1,859 EM images and 11,755 terminals in four control rats and 1,672 EM images and 8,897 terminals in four stressed rats (**Tables 1**, **2**). On average, we analyzed 65–80 EM images in each cortical layer in each animal (**Table 1**). We quantified the following parameters: the total number and density of asymmetric and symmetric synaptic terminals (n/μm^3^); the average axon terminal membrane length (nm); average synapse membrane length (nm) and the total number and density of myelinated axons (n/μm^3^). We measured these values in the six cortical layers separately. Values obtained from the individual rats are shown in **Table 1**.

**Table 2:** Results of quantitative EM analysis: Group values.

	Control	Stress	Statistical analysis
Total number of analyzed sample areas^∗^	1,859	1,672	–
Total number of analyzed axon terminals	11,755	8,897	–
Density of all axon terminals (n/μm^3^)	5.97 ± 0.27	5.91 ± 0.26	n.s.
Total number of asymmetric synapses	(1.15 ± 0.04) × 10^9^	(0.98 ± 0.04) × 10^9^	*t* = 2.84, *P* < 0.05
Density of asymmetric synapses (n/μm^3^)	5.46 ± 0.22	5.44 ± 0.25	n.s.
Total number of symmetric synapses	(1.06 ± 0.11) × 10^8^	(0.83 ± 0.06) × 10^8^	*t* = 1.77, *P* = 0.06
Density of symmetric synapses (n/μm^3^)	0.50 ± 0.06	0.46 ± 0.04	n.s.
Terminal membrane length (nm)	488.68 ± 11.20	482.07 ± 12.55	n.s.
Synapse membrane length (nm)	207.17 ± 1.74	212.21 ± 3.40	n.s.
Total number of myelinated axons	(1.00 ± 0.10) × 10^8^	(0.68 ± 0.04) × 10^8^	*t* = 2.63, *P* < 0.05
Density of myelinated axons (n/μm^3^)	0.47 ± 0.05	0.38 ± 0.03	n.s.


*Data are expressed as mean ± SEM. The total synapse numbers were calculated by multiplying synapse densities with the volume of the IL cortex. ^∗^Each sample area was 14.995 μm^*2*^. n.s., Non-significant difference.*

### Synapse Numbers in the IL Cortex of Control Rats

**FIGURE 4:**
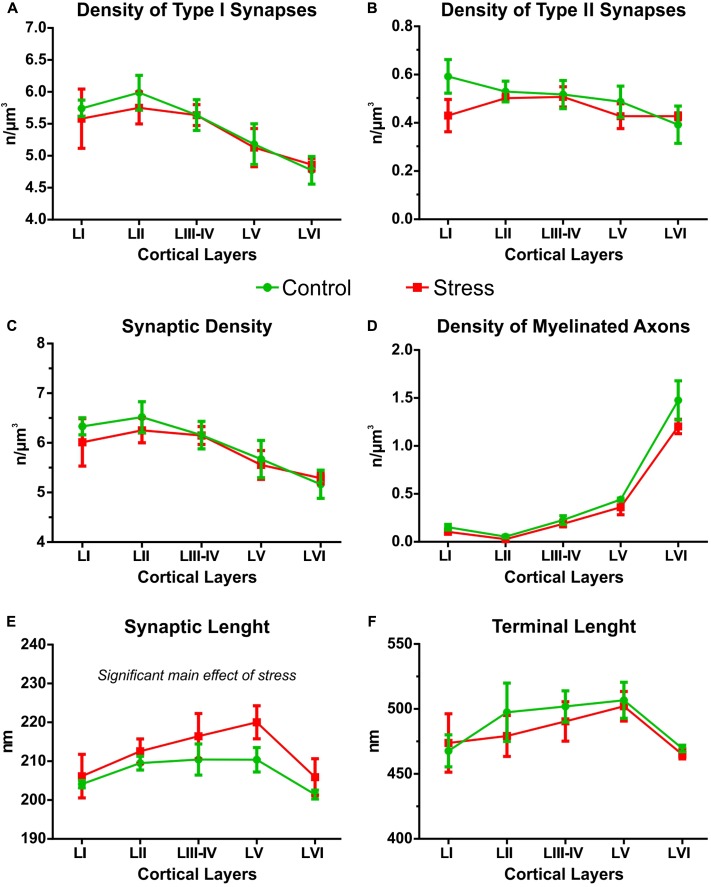
Cortical layer specific distribution of synaptic contacts in the IL cortex. All data shown in this figure were analyzed with two-way ANOVA (stress × cortical layer). **(A)** The density of Type I synapses was always higher in the upper cortical layers (I–III) but stress had no effect on this. **(B)** The distribution of Type II synapses was equal in all cortical layers. Stress had no effect on this parameter. **(C)** Synaptic densities (Type I and Type II) were always higher in the upper cortical layers (I–III), and stress had no effect on this. **(D)** The density of myelinated axons was higher in the lower cortical layers, especially in layer VI, and stress had no effect on this parameter. **(E)** Synaptic lengths were different in the various cortical layers. In the stressed rats synaptic lengths were longer compared to controls. **(F)** Axon terminal lengths were similar in all cortical layers and stress had no effect on this parameter.

The summary of our data is shown in **Tables 1**, **2**. In control rats, we observed the following values: the density of all synaptic terminals was 5.97 ± 0.27/μm^3^. The total number of asymmetric synapses was 1.15 × 10^9^ and the density of asymmetric synaptic terminals was 5.46 ± 0.22/μm^3^. The total number of symmetric synapses was 1.06 × 10^8^ and the density of symmetric synaptic terminals was 0.50 ± 0.06/μm^3^. Synaptic densities of asymmetric synapses were always higher in cortical layers I–IV compared to the deeper layers (**Figure 4A**). In contrast, the distribution of inhibitory synapses was equal within the six layers (**Figure 4B**). The average synaptic membrane length was 207.2 ± 1.8 nm and the average axon terminal length was 488.7 ± 11.2 nm. Synapse and axon terminal lengths showed a minimal (5–8%) variation within the six cortical layers (**Figures 4E,F**). The synapse/terminal ratio was 52.7 ± 0.9% and we found 116.3 ± 2.0 synapses in 100 axon terminals. We also quantified the density of myelinated axons in the neuropil and on average this number was 0.47 ± 0.05/μm^3^. The number of myelinated axons in the IL was and 1.00 × 10^8^. The number of myelinated axons varied greatly within the cortical layers, being highest in layer VI (**Figure 4D**).

### Synaptic Parameters in the Different Cortical Layers

Theoretically, it may happen that stress has a layer specific effect and alters synapse numbers only in specific cortical layers. Therefore, we compared the cortical layer specific distribution of synaptic contacts in the control and stressed rats with two-way ANOVA (stress × cortical layer, see **Figure 4**). The density of Type I synapses was always higher in the upper cortical layers [two-way ANOVA showed significant main effect of cortical layers *F*_(4,30)_ = 5.29, *P* < 0.005]. Stress exposure had no effect on the cortical distribution of Type I synaptic densities (**Figure 4A**). The distribution of Type II synapses was equal in all cortical layers and stress had no effect on this parameter (**Figure 4A**). The density of all synapses (Type I and Type II) was always higher in the upper cortical layers [significant main effect of cortical layers *F*_(4,30)_ = 5.38, *P* < 0.005] and stress had no effect on that (**Figure 4C**). The density of myelinated axons was higher in the lower cortical layers, especially in layer VI [significant main effect of cortical layers *F*_(4,30)_ = 97.14, *P* < 0.0001], but again stress had no effect on this parameter (**Figure 4D**). Synaptic lengths were different in the six cortical layers and stress also had a significant effect on synaptic length. Two-way ANOVA showed significant main effect of cortical layers [*F*_(4,30)_ = 3.51, *P* < 0.05] and significant main effect of stress [*F*_(1,30)_ = 4.32, *P* < 0.05] and no interaction. This finding indicates that in the stressed rats, synaptic lengths were always longer in each cortical layer compared to the controls (**Figure 4E**). Axon terminal lengths were similar in all cortical layers and stress had no effect on this parameter (**Figure 4F**).

### Stress Reduced the Volume of the IL Cortex

**FIGURE 5:**
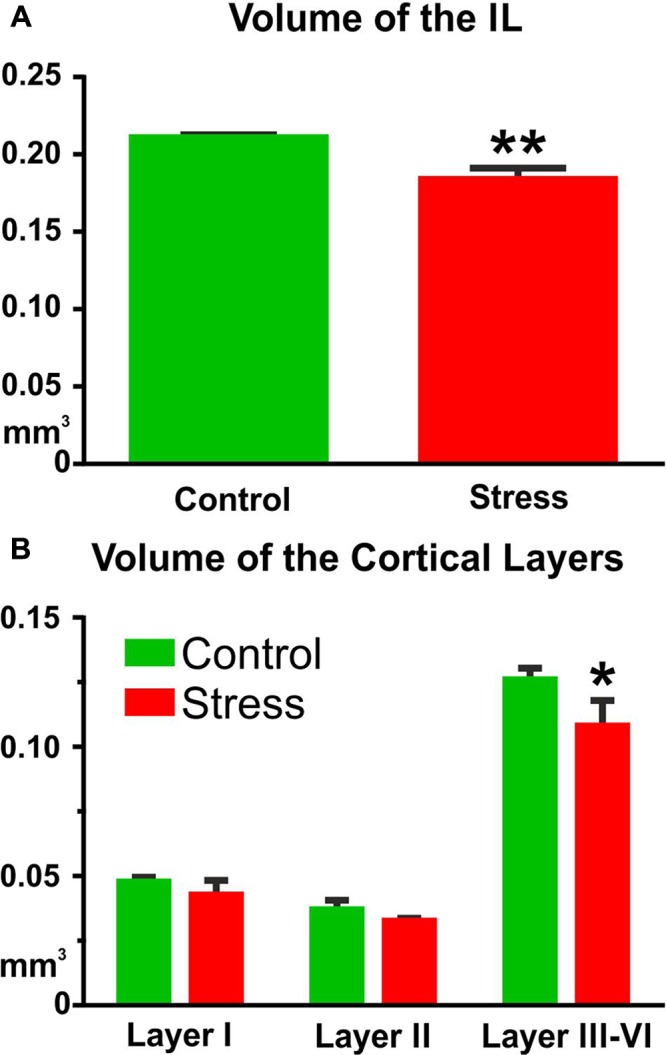
Stress reduced the volume of the IL cortex. Cavalieri’s principle was used for volume measurements. **(A)** The total volume of the IL was significantly reduced in the stressed animals (^∗∗^*P* < 0.01). **(B)** Layer specific analysis revealed that this volume shrinkage was mainly due to the volume reduction of the deeper cortical layers (III–VI). Two-way ANOVA (stress × cortical layer) revealed significant main effect of stress (*P* < 0.001) and Sidak’s *post hoc* analysis found significant difference between the control and stress groups in cortical layers III–VI (^∗^*P* < 0.001).

In control rats, the volume of the IL cortex was 0.211 ± 0.002 mm^3^ (**Figure 5**). In the stressed animals, the volume of the IL cortex was reduced to 0.184 ± 0.007 mm^3^ (*t*-test: *t* = 3.61, *P* < 0.01, **Figure 5A**). Comparison of the volumes of the different cortical layers with two-way ANOVA (stress × cortical layer) revealed significant main effect of stress [*F*_(1,18)_ = 17.75, *P* < 0.001] and significant main effect of cortical layers [*F*_(2,18)_ = 575.6, *P* < 0.001] as well as significant interaction between the two factors [*F*_(2,18)_ = 4.15, *P* < 0.05]. *Post hoc* analysis with Sidak’s multiple comparison revealed that stress reduced the volume of layers III–VI (*t* = 4.78, *P* < 0.001, **Figure 5B**).

### Stress Reduced the Number of Synapses in the IL Cortex

**FIGURE 6:**
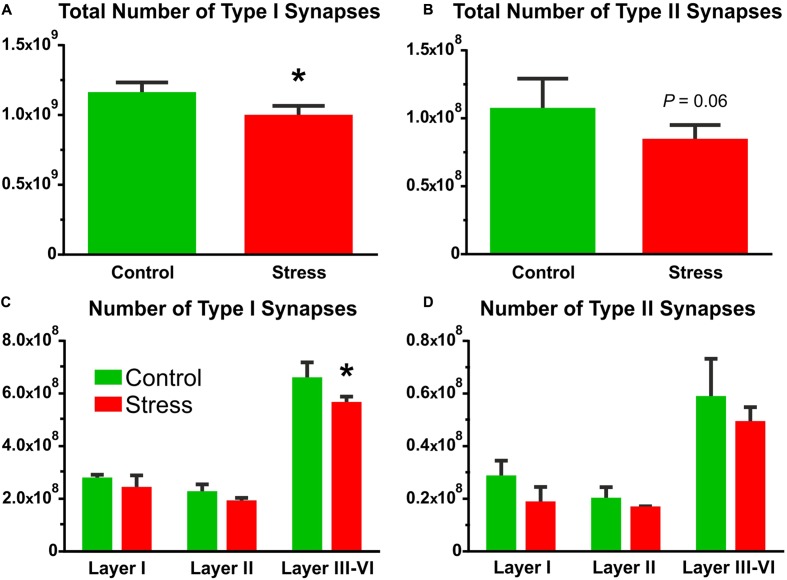
The number of Type I and Type II synapses in the IL cortex. Synapse numbers were calculated by multiplying synapse densities with the volume of the IL cortex. **(A)** Stress significantly reduced the number of asymmetric synapses (*t*-test, ^∗^*P* < 0.05). **(B)** Symmetric synapse numbers were also reduced in the stressed rats and the difference approached the level of significance (*t*-test, *P* = 0.06). **(C)** Comparison of Type I synapse numbers in the different cortical layers with two-way ANOVA (stress × cortical layer) revealed significant main effect of stress (*P* < 0.01), and significant main effect of cortical layers (*P* < 0.0001). Sidak’s *post hoc* analysis yielded that stress reduced Type I synapse numbers in layers III–VI (^∗^*P* < 0.05). **(D)** Comparison of Type II synapse numbers in the different cortical layers with two-way ANOVA (stress × cortical layer) revealed significant main effect of stress (*P* < 0.05), and significant main effect of cortical layers (*P* < 0.0001). But the *post hoc* analysis found no difference between the groups.

Total synapse numbers were calculated by multiplying synapse densities with the volume of the IL cortex (volume × density = total synapse number). The individual and group values of the total synapse numbers are displayed in **Tables 1**, **2** and **Figure 6**.

In control rats, the number of asymmetric synapses was (1.15 ± 0.04) × 10^9^ and stress reduced this number to (0.98 ± 0.04) × 10^9^ (*t*-test: *t* = 2.84, *P* < 0.05, **Figure 6A**). The number of symmetric synapses was (1.06 ± 0.11) × 10^8^ in the control rats and stress reduced this number to (0.83 ± 0.06) × 10^8^ (*t*-test: *t* = 1.77, *P* = 0.06, **Figure 6B**).

Comparison of the asymmetric synapse numbers in the different cortical layers with two-way ANOVA (stress × cortical layer) revealed significant main effect of stress [*F*_(1,18)_ = 11.63, *P* < 0.01], and significant main effect of cortical layers [*F*_(2,18)_ = 252.6, *P* < 0.0001], but no interaction between the two factors. *Post hoc* analysis with Sidak’s multiple comparison revealed that stress reduced Type I synapse numbers in the deeper cortical layers III–VI (*t* = 3.37, *P* < 0.05, **Figure 6C**).

Comparison of the symmetric synapse numbers in the different cortical layers with two-way ANOVA (stress × cortical layer) revealed significant main effect of stress [*F*_(1,18)_ = 5.82, *P* < 0.05], and significant main effect of cortical layers [*F*_(2,18)_ = 49.5, *P* < 0.0001], but no interaction between the two factors. *Post hoc* analysis with Sidak’s multiple comparison found no significant difference between the two groups (**Figure 6D**).

We should add here that we did not find any indication of neuronal degeneration or cell death in the IL cortex of the stressed animals.

### Stress Reduced the Number of Myelinated Axons in the IL Cortex

The number of myelinated axons were calculated by multiplying axon densities with the volume of the IL cortex (volume × density = total myelinated axon number).

**FIGURE 7:**
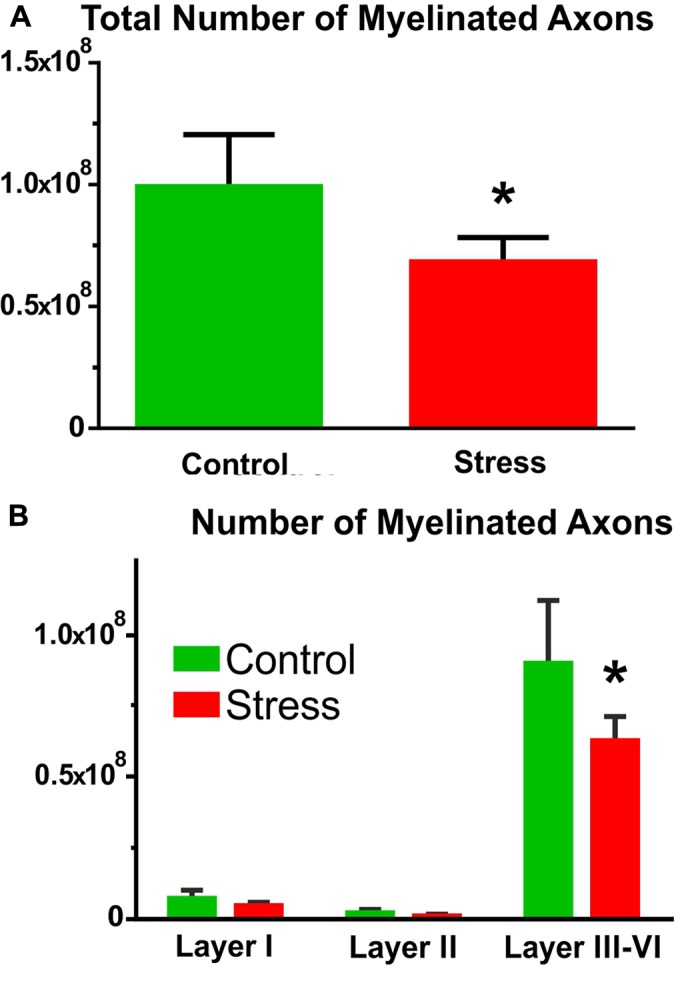
The number of myelinated axons in the IL cortex. **(A)** Stress significantly reduced the number of myelinated axons (*t*-test, ^∗^*P* < 0.05). **(B)** Comparison of axon numbers in the different cortical layers with two-way ANOVA (stress × cortical layer) revealed significant main effect of stress and of cortical layers as well as significant interaction between the two factors. Sidak’s *post hoc* analysis found that stress reduced axon numbers in cortical layers III–VI (^∗^*P* < 0.01).

In control rats, the number of myelinated axons was (1.00 ± 0.10) × 10^8^ and stress significantly reduced this number to (0.68 ± 0.04) × 10^8^ (*t*-test: *t* = 2.63, *P* < 0.05, **Figure 7A**).

Comparison of the myelinated axon numbers in the different cortical layers with two-way ANOVA (stress × cortical layer) revealed significant main effect of stress [*F*_(1,18)_ = 6.71, *P* = 0.01], significant main effect of cortical layers [*F*_(2,18)_ = 147.6, *P* < 0.0001] and significant interaction between the two factors [*F*_(2,18)_ = 4.53, *P* < 0.05]. Sidak’s *post hoc* analysis revealed that stress reduced myelinated axon numbers in cortical layers III–VI (*t* = 3.95, *P* < 0.01, **Figure 7B**).

## Discussion

To our best of knowledge this is the first detailed EM analysis of synapse numbers in the mPFC of rats subjected to long term stress. We focused on the IL cortex since this sub-area of the mPFC appears to be the most susceptible to the effect of stress. Stress significantly reduced the number of synapses and myelinated axons in the deeper cortical layers. Notably, stress had no effect on synaptic and axonal densities, but when we calculated their total numbers in the IL, then, a significant difference emerged between the control and stressed animals. Furthermore, we found that synaptic membrane lengths were increased in the stressed rats, probably to compensate the loss of synapse numbers. In sum, our present report is the first to provide ultrastructural evidence for stress-induced synapse loss in the mPFC.

**FIGURE 8:**
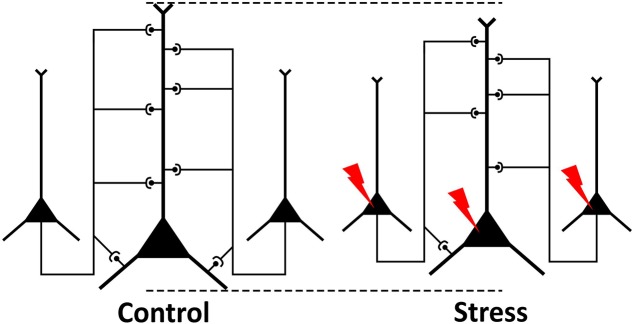
Schematic drawing illustrating the present findings. Pyramidal neurons of the neocortex communicate with each other via synapses. In stressed animals, neurons have reduced dendritic tree and some connections between the neurons are lost. Our present ultrastructural data on stress-induced loss of axons and synapses indicate that stressed neurons have reduced intra- and inter-cortical connectivity with other neurons.

Numerous studies have demonstrated that stress can induce dendritic atrophy of layer II–III pyramidal neurons in the IL cortex (Radley et al., 2004; Izquierdo et al., 2006; Perez-Cruz et al., 2007, 2009; Dias-Ferreira et al., 2009; Goldwater et al., 2009; Shansky et al., 2009). Our present data complement and extend these findings by providing ultrastructural evidences on reduced axon numbers and loss of axo-spinous excitatory synapses. This data demonstrates that the intra- and inter-cortical connectivity of neurons in the IL cortex is reduced (**Figure 8**). This finding is in harmony with the recent evidences that stress can affect white matter integrity and myelinated fibers (Miyata et al., 2016; Gao et al., 2017; Xiao et al., 2018). Reduced network connectivity of neurons may form the anatomical basis for impaired functioning of this brain area (Menon, 2011). It is known that synapse loss is present in several brain areas of patients with neurodegenerative disorders like Alzheimer’s dementia and the synapse loss correlates well with the cognitive decline (e.g., Minger et al., 2001; Clare et al., 2010; Robinson et al., 2014). Loss of asymmetric synapses has also been found in the prefrontal cortex of cognitively impaired monkeys in a primate model for Parkinson’s disease (Elsworth et al., 2013), suggesting that the synapse loss in the PFC was responsible for the cognitive deficits. Cognitive deficits are common in depressed patients (Lam et al., 2014) as well as in stressed individuals (Jonsdottir et al., 2013) and synapse loss in the prefrontal cortex is likely to contribute to these cognitive impairments.

Synaptic contacts are key functional and structural elements of the central nervous system, since proper synaptic transmission is essential for normal nervous system function. Synapse numbers and morphology are important neuroanatomical data both in the healthy and diseased brain. Numerous studies documented pronounced remodeling of excitatory spine synapses in the hippocampi of stressed animals (e.g., Magariños et al., 1997; Sandi et al., 2003; Stewart et al., 2005; Donohue et al., 2006; Hajszan et al., 2009). Similar structural changes are expected to take place in the prefrontal cortex (Musazzi et al., 2015), but only few studies investigated that. For example, a recent EM study investigated morphological remodeling of asymmetric synapses in the anterior cingulate cortex of rats subjected to chronic unpredictable mild stress (Li et al., 2015). They found a considerable remodeling of synapses including changes in width of the synaptic cleft, length of the active zones and postsynaptic density thickness (Li et al., 2015), but they did not report on synapse numbers. Another study investigated synapses in the IL cortex of rats bred for learned helplessness (Seese et al., 2013), which is a rat model for congenital depression. They found that synapses immunolabeled for the postsynaptic density marker PSD-95 had the same numerical density, but lower immunolabeling intensities (Seese et al., 2013).

The above described neuroanatomical changes are complemented by functional studies which reveal altered or disturbed excitatory neurotransmission in the mPFC of stressed animals. For example reduced AMPA and NMDA receptor-mediated synaptic transmission and reduced glutamate receptor expression were found in the mPFC of stressed animals (Yuen et al., 2012). Reduced activity of glutamatergic and GABAergic neurons were observed in stressed mice having a depressive-like phenotype (Veeraiah et al., 2014). Others found diminished responses to apically targeted excitatory inputs in layer V pyramidal neurons (Liu and Aghajanian, 2008) and impaired LTP formation in the IL cortex (Goldwater et al., 2009). Wilber et al. (2011) found altered neuronal activity in the stressed mPFC during fear conditioning and extinction. Yet another group reported that excitatory synaptic potentiation in the mPFC was linked to learned helplessness (a depressive-like behavior) whereas, synaptic weakening was associated with resilience to stress (Wang et al., 2014).

Recent theories emphasize the importance of disturbed neuroplasticity in the pathophysiology of depressive disorders (e.g., Pittenger and Duman, 2008; Castrén, 2013). These theories are based on the clinical findings documenting functional abnormalities in the prefrontal cortex of depressed patients (e.g., Baxter et al., 1989; Drevets et al., 1997; Mayberg et al., 1999), as well as volume shrinkage of various fronto-cortical areas (Drevets et al., 1998, 2008; Rajkowska et al., 1999; Price and Drevets, 2010). This neuroplasticity-theory of depression argues that at the synaptic level, the stress-induced structural and functional changes of excitatory synapses are the key contributors to the pathophysiology (Popoli et al., 2011; Duman and Aghajanian, 2012; Licznerski and Duman, 2013; Timmermans et al., 2013; Duman, 2014; Thompson et al., 2015; Duman et al., 2016). Supporting this theory, a recent post-mortem EM study demonstrated lower number of synapses in the dorsolateral prefrontal cortex of patients with major depressive disorder (Kang et al., 2012). It has been argued that the disturbed synaptic communication results in disrupted circuitry within and between specific cortico-limbic structures and these are the key contributors to the disturbed emotional behavior of depressive syndromes. Furthermore, such stress-induced structural abnormalities of cortical networks are likely to contribute also to the cognitive deficits commonly observed in depressed patients (Austin et al., 2001; Lam et al., 2014), stressed individuals (Soares et al., 2012; Jonsdottir et al., 2013; Arnsten, 2015) and in other psychiatric disorders, like schizophrenia (Arnsten, 2011). Our present finding on reduced synapse numbers in the mPFC of rats clearly supports these theories. Obviously, it is very difficult – if not impossible – to identify the exact human analogy of the rodent IL cortex, but most likely similar structural changes take place in the stressed human frontal cortex.

There are different methods to quantify the number and morphology of synaptic contacts in the CNS. One can do immunolabeling of synaptic proteins and then, do quantitative light microscopic analysis (e.g., Seese et al., 2013; Drzewiecki et al., 2016). However, because of the small size of the synapses, EM examination is still regarded as the gold standard for investigating synaptic contacts (DeFelipe et al., 1999). DeFelipe et al. (1999) compared the two most commonly used synapse quantification methods, the size-frequency versus the disector method. Because the disector method is considered to be an unbiased method, thus, typically this method is recommended for the quantification of synapses in the neocortex. DeFelipe et al. (1999) demonstrated that in fact the two methods yield similar estimates for the numerical density of synapses and they also proved that the size-frequency method is more efficient and easier to apply than the disector method. Therefore, we applied the size-frequency method in our present study. First, we quantified synaptic densities in the six cortical layers and we found very similar values both in the control and stressed rats. The stress-induced difference emerged only when we measured the volume of the IL cortex and combined the volumetric data with the synaptic densities. Stress-induced volume loss of the mPFC has been documented by other groups as well (e.g., Cerqueira et al., 2007; Dias-Ferreira et al., 2009). We also found an increased length of synapses which may indicate a compensatory rearrangement of synaptic contacts. Notably, similar synaptic changes – loss of number, but increased length – have been observed in frontal brain samples from patients with Alzheimer’s disease (DeKosky and Scheff, 1990).

Here, we quantified the number of synapses and axons in the six cortical layers separately. The density of asymmetric synapses was always higher in the upper cortical layers (I–III) while the distribution of inhibitory synapses was equal within the six cortical layers (**Figure 4**). In contrast, axon numbers were high in the deep cortical layers (V–VI). These data are in harmony with the findings of other cortical areas (see e.g., DeFelipe et al., 1999, 2002; Douglas et al., 2004; DeFelipe, 2011; Anton-Sanchez et al., 2014). Stress had no effect on these density parameters. However, a different picture emerged when we combined the density data with the volume measurements. We measured the volume of the IL cortex which enabled us to report on the total number of axons and synapses in the IL cortex. During the volume measurements, we aimed to differentiate between the six cortical layers using the description of Gabbott et al. (1997). It was easy to differentiate cortical layer I, because this layer contains only a few scattered neurons. It was also easy to define layer II, because this layer has higher density of cell bodies compared to the deeper layers. However, in the IL cortex, it is not easy to see the borders between layers III–VI, therefore, we decided to group them together (**Figure 5B**). Collectively, these four layers made up about two-third (65%) of the entire cortical volume. In the stressed rats, every cortical layer became thinner, but the volume shrinkage was the largest in the combined layers of III–VI (**Figure 5B**). The *post hoc* analysis of the two-way ANOVA found a significant difference between the groups only in these deeper layers. The two-way ANOVA (stress × cortical layer) also found a significant interaction between the two factors because the effect of the two factors were pointing into an opposite directions. Stress reduced the volume of the cortical layers while volume in the deeper layers was increasing because there we grouped four layers together. When we multiplied the cortical volume data with the synapse and axon density values the volumetric changes determined the changes in the total number of synapses and axons (**Figures 5**–**7**). For this reason, we found significant reductions in axon and synapse numbers only in the deeper layers (III–VI). Two-way ANOVA analysis (stress × cortical layer) found also significant interaction between the two factors in case of the axon number data. The reason for this interaction was that the effect of stress treatment was the greatest in the deeper cortical layers, where axons were also much more numerous compared to the higher cortical layers.

Our present finding on stress-induced loss of synapses in the deep layers of the PFC is in harmony with the light microscopy data, which documents loss of dendritic material in the same region. The light microscopy studies, investigating the effect of stress on dendritic architecture, typically focus on pyramidal neurons of layer III. In the IL cortex, stress-induced loss of dendritic length has been found, either in the proximal apical dendritic region (10–100 μm from soma) (Perez-Cruz et al., 2007), or in the intermediate apical dendritic region (120–180 μm from soma) (Shansky et al., 2009). Furthermore, it has been shown that stress can also reduce the length of basilar dendrites of layer III pyramidal neurons (Perez-Cruz et al., 2009). The same study also documented that the stress-induced loss of dendritic spines was present only in the proximal region of the apical dendritic tree (0–30 μm from the soma) (Perez-Cruz et al., 2009). In sum, these studies documented stress-induced loss of dendritic material in the deeper cortical layers (layer III–VI) where we also found a significant loss of synaptic contacts (**Figure 8**) and reduction of cortical volume.

Finally, we should state that in our ultrastructural analysis, we found no evidence of neuronal degeneration or cell death in the IL cortex of the stressed animals. We should add, however, that our histopathological analysis was done only once, at the end of the 9-weeks of the stress protocol and cells could have been lost without trace at earlier stages of the stress procedure, since apoptosis is a rapid cellular process.

In summary, we report here that experimentally induced long term stress can reduce the number of asymmetric synapses and myelinated axons in the mPFC of rats. Similar loss of synaptic contacts may occur in humans and could contribute to the cognitive deficits frequently observed in stressed individuals and depressed patients.

## Author Contributions

The contributions were the following: DC did all the quantitative electron microscopic analysis and prepared the figures for the paper; OW designed the experiment and provided supervision; BC had the experimental idea, designed the experiment and wrote the paper. All authors have approved the final version of article.

## Conflict of Interest Statement

The authors declare that the research was conducted in the absence of any commercial or financial relationships that could be construed as a potential conflict of interest.

## References

[B1] Anton-Sanchez L. Bielza C. Merchán-Pérez A. Rodríguez J. R. DeFelipe J. Larrañaga P. (2014). Three-dimensional distribution of cortical synapses: a replicated point pattern-based analysis. *Front. Neuroanat.* 8:85. 10.3389/fnana.2014.00085 25206325 PMC4143965

[B2] Arnsten A. F. (2011). Prefrontal cortical network connections: key site of vulnerability in stress and schizophrenia. *Int. J. Dev. Neurosci.* 29 215–223. 10.1016/j.ijdevneu.2011.02.006 21345366 PMC3115784

[B3] Arnsten A. F. (2015). Stress weakens prefrontal networks: molecular insults to higher cognition. *Nat. Neurosci.* 18 1376–1385. 10.1038/nn.4087 26404712 PMC4816215

[B4] Austin M. P. Mitchell P. Goodwin G. M. (2001). Cognitive deficits in depression: possible implications for functional neuropathology. *Br. J. Psychiatry* 178 200–206. 10.1192/bjp.178.3.200 11230029

[B5] Baka J. Csakvari E. Huzian O. Dobos N. Siklos L. Leranth C. (2017). Stress induces equivalent remodeling of hippocampal spine synapses in a simulated postpartum environment and in a female rat model of major depression. *Neuroscience* 343 384–397. 10.1016/j.neuroscience.2016.12.021 28012870 PMC5421158

[B6] Baxter L. R. Jr. Schwartz J. M. Phelps M. E. Mazziotta J. C. Guze B. H. Selin C. E. (1989). Reduction of prefrontal cortex glucose metabolism common to three types of depression. *Arch. Gen. Psychiatry* 46 243–250. 10.1001/archpsyc.1989.01810030049007 2784046

[B7] Castrén E. (2013). Neuronal network plasticity and recovery from depression. *JAMA Psychiatry* 70 983–989. 10.1001/jamapsychiatry.2013.1 23842648

[B8] Cerqueira J. J. Mailliet F. Almeida O. F. Jay T. M. Sousa N. (2007). The prefrontal cortex as a key target of the maladaptive response to stress. *J. Neurosci.* 27 2781–2787. 10.1523/JNEUROSCI.4372-06.2007 17360899 PMC6672565

[B9] Clare R. King V. G. Wirenfeldt M. Vinters H. V. (2010). Synapse loss in dementias. *J. Neurosci. Res.* 88 2083–2090. 10.1002/jnr.22392 20533377 PMC3068914

[B10] Cook S. C. Wellman C. L. (2004). Chronic stress alters dendritic morphology in rat medial prefrontal cortex. *J. Neurobiol.* 60 236–248. 10.1002/neu.20025 15266654

[B11] Csabai D. Seress L. Varga Z. Ábrahám H. Miseta A. Wiborg O. (2017). Electron microscopic analysis of hippocampal axo-somatic synapses in a chronic stress model for depression. *Hippocampus* 27 17–27. 10.1002/hipo.22650 27571571 PMC5215622

[B12] Czéh B. Fuchs E. Wiborg O. Simon M. (2016). Animal models of major depression and their clinical implications. *Prog. Neuropsychopharmacol. Biol. Psychiatry* 64 293–310. 10.1016/j.pnpbp.2015.04.004 25891248

[B13] Dalley J. W. Cardinal R. N. Robbins T. W. (2004). Prefrontal executive and cognitive functions in rodents: neural and neurochemical substrates. *Neurosci. Biobehav. Rev.* 28 771–784. 10.1016/j.neubiorev.2004.09.006 15555683

[B14] DeFelipe J. (2011). The evolution of the brain, the human nature of cortical circuits, and intellectual creativity. *Front. Neuroanat.* 5:29. 10.3389/fnana.2011.00029 21647212 PMC3098448

[B15] DeFelipe J. Alonso-Nanclares L. Arellano J. I. (2002). Microstructure of the neocortex: comparative aspects. *J. Neurocytol.* 31 299–316. 10.1023/A:1024130211265 12815249

[B16] DeFelipe J. Marco P. Busturia I. Merchán-Pérez A. (1999). Estimation of the number of synapses in the cerebral cortex: methodological considerations. *Cereb. Cortex* 9 722–732. 10.1093/cercor/9.7.722 10554995

[B17] DeKosky S. T. Scheff S. W. (1990). Synapse loss in frontal cortex biopsies in Alzheimer’s disease: correlation with cognitive severity. *Ann. Neurol.* 27 457–464. 10.1002/ana.410270502 2360787

[B18] Dias-Ferreira E. Sousa J. C. Melo I. Morgado P. Mesquita A. R. Cerqueira J. J. (2009). Chronic stress causes frontostriatal reorganization and affects decision-making. *Science* 325 621–625. 10.1126/science.1171203 19644122

[B19] Donohue H. S. Gabbott P. L. Davies H. A. Rodríguez J. J. Cordero M. I. Sandi C. (2006). Chronic restraint stress induces changes in synapse morphology in stratum lacunosum-moleculare CA1 rat hippocampus: a stereological and three-dimensional ultrastructural study. *Neuroscience* 140 597–606. 10.1016/j.neuroscience.2006.02.072 16600515

[B20] Douglas R. Markram H. Martin K. (2004). “Neocortex,” in *The Synaptic Organization of the Brain*, 5th Edn, ed. Shepherd G. M. (Oxford: Oxford University Press), 499–558. 10.1093/acprof:oso/9780195159561.003.0012

[B21] Drevets W. C. Ongür D. Price J. L. (1998). Neuroimaging abnormalities in the subgenual prefrontal cortex: implications for the pathophysiology of familial mood disorders. *Mol. Psychiatry* 3 220–226. 10.1038/sj.mp.4000370 9672897

[B22] Drevets W. C. Price J. L. Furey M. L. (2008). Brain structural and functional abnormalities in mood disorders: implications for neurocircuitry models of depression. *Brain Struct. Funct.* 213 93–118. 10.1007/s00429-008-0189-x 18704495 PMC2522333

[B23] Drevets W. C. Price J. L. Simpson J. R. Jr. Todd R. D. Reich T. Vannier M. (1997). Subgenual prefrontal cortex abnormalities in mood disorders. *Nature* 386 824–827. 10.1038/386824a0 9126739

[B24] Drzewiecki C. M. Willing J. Juraska J. M. (2016). Synaptic number changes in the medial prefrontal cortex across adolescence in male and female rats: a role for pubertal onset. *Synapse* 70 361–368. 10.1002/syn.21909 27103097 PMC4945496

[B25] Duman R. S. (2014). Pathophysiology of depression and innovative treatments: remodeling glutamatergic synaptic connections. *Dialogues Clin. Neurosci.* 16 11–27. 24733968 10.31887/DCNS.2014.16.1/rduman PMC3984887

[B26] Duman R. S. Aghajanian G. K. (2012). Synaptic dysfunction in depression: potential therapeutic targets. *Science* 338 68–72. 10.1126/science.1222939 23042884 PMC4424898

[B27] Duman R. S. Aghajanian G. K. Sanacora G. Krystal J. H. (2016). Synaptic plasticity and depression: new insights from stress and rapid-acting antidepressants. *Nat. Med.* 22 238–249. 10.1038/nm.4050 26937618 PMC5405628

[B28] Elsworth J. D. Leranth C. Redmond D. E. Jr. Roth R. H. (2013). Loss of asymmetric spine synapses in prefrontal cortex of motor-asymptomatic, dopamine-depleted, cognitively impaired MPTP-treated monkeys. *Int. J. Neuropsychopharmacol.* 16 905–912. 10.1017/S1461145712000892 22947206 PMC3733504

[B29] Flak J. N. Solomon M. B. Jankord R. Krause E. G. Herman J. P. (2012). Identification of chronic stress-activated regions reveals a potential recruited circuit in rat brain. *Eur. J. Neurosci.* 36 2547–2555. 10.1111/j.1460-9568.2012.08161.x 22789020 PMC4538599

[B30] Gabbott P. L. Dickie B. G. Vaid R. R. Headlam A. J. Bacon S. J. (1997). Local-circuit neurones in the medial prefrontal cortex (areas 25, 32 and 24b) in the rat: morphology and quantitative distribution. *J. Comp. Neurol.* 377 465–499. 10.1002/(SICI)1096-9861(19970127)377:4<465::AID-CNE1>3.0.CO;2-0 9007187

[B31] Gao Y. Ma J. Tang J. Liang X. Huang C. X. Wang S. R. (2017). White matter atrophy and myelinated fiber disruption in a rat model of depression. *J. Comp. Neurol.* 525 1922–1933. 10.1002/cne.24178 28118485

[B32] Goldwater D. S. Pavlides C. Hunter R. G. Bloss E. B. Hof P. R. McEwen B. S. (2009). Structural and functional alterations to rat medial prefrontal cortex following chronic restraint stress and recovery. *Neuroscience* 164 798–808. 10.1016/j.neuroscience.2009.08.053 19723561 PMC2762025

[B33] Gundersen H. J. Bagger P. Bendtsen T. F. Evans S. M. Korbo L. Marcussen N. (1988). The new stereological tools: disector, fractionator, nucleator and point sampled intercepts and their use in pathological research and diagnosis. *APMIS* 96 857–881. 10.1111/j.1699-0463.1988.tb00954.x 3056461

[B34] Gundersen H. J. G. (1977). Notes on the estimation of the numerical density of arbitrary profiles: the edge effect. *J. Microsc.* 111 219–223. 10.1111/j.1365-2818.1977.tb00062.x

[B35] Hajszan T. Dow A. Warner-Schmidt J. L. Szigeti-Buck K. Sallam N. L. Parducz A. (2009). Remodeling of hippocampal spine synapses in the rat learned helplessness model of depression. *Biol. Psychiatry* 65 392–400. 10.1016/j.biopsych.2008.09.031 19006787 PMC2663388

[B36] Helmeke C. Ovtscharoff W. Jr. Poeggel G. Braun K. (2001). Juvenile emotional experience alters synaptic inputs on pyramidal neurons in the anterior cingulate cortex. *Cereb. Cortex* 11 717–727. 10.1093/cercor/11.8.717 11459761

[B37] Henningsen K. Andreasen J. T. Bouzinova E. V. Jayatissa M. N. Jensen M. S. Redrobe J. P. (2009). Cognitive deficits in the rat chronic mild stress model for depression: relation to anhedonic-like responses. *Behav. Brain Res.* 198 136–141. 10.1016/j.bbr.2008.10.039 19038290

[B38] Henningsen K. Palmfeldt J. Christiansen S. Baiges I. Bak S. Jensen O. N. (2012). Candidate hippocampal biomarkers of susceptibility and resilience to stress in a rat model of depression. *Mol. Cell. Proteomics* 11:M111.016428. 10.1074/mcp.M111.016428 22311638 PMC3394954

[B39] Hinwood M. Tynan R. J. Day T. A. Walker F. R. (2011). Repeated social defeat selectively increases δFosB expression and histone H3 acetylation in the infralimbic medial prefrontal cortex. *Cereb. Cortex* 21 262–271. 10.1093/cercor/bhq080 20513656

[B40] Holmes A. Wellman C. L. (2009). Stress-induced prefrontal reorganization and executive dysfunction in rodents. *Neurosci. Biobehav. Rev.* 33 773–783. 10.1016/j.neubiorev.2008.11.005 19111570 PMC2941982

[B41] Izquierdo A. Wellman C. L. Holmes A. (2006). Brief uncontrollable stress causes dendritic retraction in infralimbic cortex and resistance to fear extinction in mice. *J. Neurosci.* 26 5733–5738. 10.1523/JNEUROSCI.0474-06.2006 16723530 PMC6675270

[B42] Jonsdottir I. H. Nordlund A. Ellbin S. Ljung T. Glise K. Währborg P. (2013). Cognitive impairment in patients with stress-related exhaustion. *Stress* 16 181–190. 10.3109/10253890.2012.708950 22746338

[B43] Kang H. J. Voleti B. Hajszan T. Rajkowska G. Stockmeier C. A. Licznerski P. (2012). Decreased expression of synapse-related genes and loss of synapses in major depressive disorder. *Nat. Med.* 18 1413–1417. 10.1038/nm.2886 22885997 PMC3491115

[B44] Koot S. Koukou M. Baars A. Hesseling P. van ’t Klooster J. Joëls M. (2014). Corticosterone and decision-making in male Wistar rats: the effect of corticosterone application in the infralimbic and orbitofrontal cortex. *Front. Behav. Neurosci.* 8:127. 10.3389/fnbeh.2014.00127 24795580 PMC4001045

[B45] Lam R. W. Kennedy S. H. Mclntyre R. S. Khullar A. (2014). Cognitive dysfunction in major depressive disorder: effects on psychosocial functioning and implications for treatment. *Can. J. Psychiatry* 59 649–654. 10.1177/070674371405901206 25702365 PMC4304584

[B46] Li X. L. Yuan Y. G. Xu H. Wu D. Gong W. G. Geng L. Y. (2015). Changed synaptic plasticity in neural circuits of depressive-like and escitalopram-treated rats. *Int. J. Neuropsychopharmacol.* 18:pyv046. 10.1093/ijnp/pyv046 25899067 PMC4648155

[B47] Licznerski P. Duman R. S. (2013). Remodeling of axo-spinous synapses in the pathophysiology and treatment of depression. *Neuroscience* 251 33–50. 10.1016/j.neuroscience.2012.09.057 23036622 PMC3566360

[B48] Liston C. Miller M. M. Goldwater D. S. Radley J. J. Rocher A. B. Hof P. R. (2006). Stress-induced alterations in prefrontal cortical dendritic morphology predict selective impairments in perceptual attentional set-shifting. *J. Neurosci.* 26 7870–7874. 10.1523/JNEUROSCI.1184-06.2006 16870732 PMC6674229

[B49] Liu R. J. Aghajanian G. K. (2008). Stress blunts serotonin- and hypocretin-evoked EPSCs in prefrontal cortex: role of corticosterone-mediated apical dendritic atrophy. *Proc. Natl. Acad. Sci. U.S.A.* 105 359–364. 10.1073/pnas.0706679105 18172209 PMC2224217

[B50] Lucassen P. J. Pruessner J. Sousa N. Almeida O. F. Van Dam A. M. Rajkowska G. (2014). Neuropathology of stress. *Acta Neuropathol.* 127 109–135. 10.1007/s00401-013-1223-5 24318124 PMC3889685

[B51] Magariños A. M. Verdugo J. M. McEwen B. S. (1997). Chronic stress alters synaptic terminal structure in hippocampus. *Proc. Natl. Acad. Sci. U.S.A.* 94 14002–14008. 10.1073/pnas.94.25.14002 9391142 PMC28422

[B52] Martin K. P. Wellman C. L. (2011). NMDA receptor blockade alters stress-induced dendritic remodeling in medial prefrontal cortex. *Cereb. Cortex* 21 2366–2373. 10.1093/cercor/bhr021 21383235 PMC3697127

[B53] Mayberg H. S. Liotti M. Brannan S. K. McGinnis S. Mahurin R. K. Jerabek P. A. (1999). Reciprocal limbic-cortical function and negative mood: converging PET findings in depression and normal sadness. *Am. J. Psychiatry* 156 675–682. 10327898 10.1176/ajp.156.5.675

[B54] McEwen B. S. Morrison J. H. (2013). The brain on stress: vulnerability and plasticity of the prefrontal cortex over the life course. *Neuron* 79 16–29. 10.1016/j.neuron.2013.06.028 23849196 PMC3753223

[B55] McKlveen J. M. Morano R. L. Fitzgerald M. Zoubovsky S. Cassella S. N. Scheimann J. R. (2016). Chronic stress increases prefrontal inhibition: a mechanism for stress-induced prefrontal dysfunction. *Biol. Psychiatry* 80 754–764. 10.1016/j.biopsych.2016.03.2101 27241140 PMC5629635

[B56] McKlveen J. M. Myers B. Flak J. N. Bundzikova J. Solomon M. B. Seroogy K. B. (2013). Role of prefrontal cortex glucocorticoid receptors in stress and emotion. *Biol. Psychiatry* 74 672–679. 10.1016/j.biopsych.2013.03.024 23683655 PMC3797253

[B57] McKlveen J. M. Myers B. Herman J. P. (2015). The medial prefrontal cortex: coordinator of autonomic, neuroendocrine and behavioural responses to stress. *J. Neuroendocrinol.* 27 446–456. 10.1111/jne.12272 25737097 PMC4580281

[B58] Menon V. (2011). Large-scale brain networks and psychopathology: a unifying triple network model. *Trends Cogn. Sci.* 15 483–506. 10.1016/j.tics.2011.08.003 21908230

[B59] Minger S. L. Honer W. G. Esiri M. M. McDonald B. Keene J. Nicoll J. A. (2001). Synaptic pathology in prefrontal cortex is present only with severe dementia in Alzheimer disease. *J. Neuropathol. Exp. Neurol.* 60 929–936. 10.1093/jnen/60.10.929 11589423

[B60] Miyata S. Taniguchi M. Koyama Y. Shimizu S. Tanaka T. Yasuno F. (2016). Association between chronic stress-induced structural abnormalities in Ranvier nodes and reduced oligodendrocyte activity in major depression. *Sci. Rep.* 6:23084. 10.1038/srep23084 26976207 PMC4791682

[B61] Moench K. M. Maroun M. Kavushansky A. Wellman C. (2015). Alterations in neuronal morphology in infralimbic cortex predict resistance to fear extinction following acute stress. *Neurobiol. Stress* 3 23–33. 10.1016/j.ynstr.2015.12.002 26844245 PMC4730795

[B62] Moghaddam B. (2002). Stress activation of glutamate neurotransmission in the prefrontal cortex: implications for dopamine-associated psychiatric disorders. *Biol. Psychiatry* 51 775–787. 10.1016/S0006-3223(01)01362-2 12007451

[B63] Musazzi L. Treccani G. Popoli M. (2015). Functional and structural remodeling of glutamate synapses in prefrontal and frontal cortex induced by behavioral stress. *Front. Psychiatry* 6:60. 10.3389/fpsyt.2015.00060 25964763 PMC4410487

[B64] Nava N. Treccani G. Liebenberg N. Chen F. Popoli M. Wegener G. (2014). Chronic desipramine prevents acute stress-induced reorganization of medial prefrontal cortex architecture by blocking glutamate vesicle accumulation and excitatory synapse increase. *Int. J. Neuropsychopharmacol.* 8:pyu085. 10.1093/ijnp/pyu085 25522419 PMC4360240

[B65] Ovtscharoff W. Jr. Braun K. (2001). Maternal separation and social isolation modulate the postnatal development of synaptic composition in the infralimbic cortex of Octodon degus. *Neuroscience* 104 33–40. 10.1016/S0306-4522(01)00059-8 11311528

[B66] Paxinos G. Watson C. (1998). *The Rat Brain in Stereotaxic Coordinates.* Cambridge, MA: Academic Press.

[B67] Perez-Cruz C. Müller-Keuker J. I. Heilbronner U. Fuchs E. Flügge G. (2007). Morphology of pyramidal neurons in the rat prefrontal cortex: lateralized dendritic remodeling by chronic stress. *Neural Plast.* 2007:46276. 10.1155/2007/46276 18253468 PMC1975761

[B68] Perez-Cruz C. Simon M. Flügge G. Fuchs E. Czéh B. (2009). Diurnal rhythm and stress regulate dendritic architecture and spine density of pyramidal neurons in the rat infralimbic cortex. *Behav. Brain Res.* 205 406–413. 10.1016/j.bbr.2009.07.021 19643147

[B69] Pittenger C. Duman R. S. (2008). Stress, depression, and neuroplasticity: a convergence of mechanisms. *Neuropsychopharmacology* 33 88–109. 10.1038/sj.npp.1301574 17851537

[B70] Popoli M. Yan Z. McEwen B. S. Sanacora G. (2011). The stressed synapse: the impact of stress and glucocorticoids on glutamate transmission. *Nat. Rev. Neurosci.* 13 22–37. 10.1038/nrn3138 22127301 PMC3645314

[B71] Price J. L. Drevets W. C. (2010). Neurocircuitry of mood disorders. *Neuropsychopharmacology* 35 192–216. 10.1038/npp.2009.104 19693001 PMC3055427

[B72] Radley J. J. Rocher A. B. Miller M. Janssen W. G. Liston C. Hof P. R. (2006). Repeated stress induces dendritic spine loss in the rat medial prefrontal cortex. *Cereb. Cortex* 16 313–320. 10.1093/cercor/bhi104 15901656

[B73] Radley J. J. Sisti H. M. Hao J. Rocher A. B. McCall T. Hof P. R. (2004). Chronic behavioral stress induces apical dendritic reorganization in pyramidal neurons of the medial prefrontal cortex. *Neuroscience* 125 1–6. 10.1016/j.neuroscience.2004.01.006 15051139

[B74] Rajkowska G. Miguel-Hidalgo J. J. Wei J. Dilley G. Pittman S. D. Meltzer H. Y. (1999). Morphometric evidence for neuronal and glial prefrontal cell pathology in major depression. *Biol. Psychiatry* 45 1085–1098. 10.1016/S0006-3223(99)00041-4 10331101

[B75] Riga D. Matos M. R. Glas A. Smit A. B. Spijker S. Van den Oever M. C. (2014). Optogenetic dissection of medial prefrontal cortex circuitry. *Front. Syst. Neurosci.* 8:230. 10.3389/fnsys.2014.00230 25538574 PMC4260491

[B76] Robinson J. L. Molina-Porcel L. Corrada M. M. Raible K. Lee E. B. Lee V. M. (2014). Perforant path synaptic loss correlates with cognitive impairment and Alzheimer’s disease in the oldest-old. *Brain* 137 2578–2587. 10.1093/brain/awu190 25012223 PMC4132652

[B77] Sandi C. Davies H. A. Cordero M. I. Rodriguez J. J. Popov V. I. Stewart M. G. (2003). Rapid reversal of stress induced loss of synapses in CA3 of rat hippocampus following water maze training. *Eur. J. Neurosci.* 17 2447–2456. 10.1046/j.1460-9568.2003.02675.x 12814376

[B78] Seese R. R. Chen L. Y. Cox C. D. Schulz D. Babayan A. H. Bunney W. E. (2013). Synaptic abnormalities in the infralimbic cortex of a model of congenital depression. *J. Neurosci.* 33 13441–13448. 10.1523/JNEUROSCI.2434-13.2013 23946402 PMC3742930

[B79] Shansky R. M. Hamo C. Hof P. R. McEwen B. S. Morrison J. H. (2009). Stress-induced dendritic remodeling in the prefrontal cortex is circuit specific. *Cereb. Cortex* 19 2479–2484. 10.1093/cercor/bhp003 19193712 PMC2742599

[B80] Soares J. M. Sampaio A. Ferreira L. M. Santos N. C. Marques F. Palha J. A. (2012). Stress-induced changes in human decision-making are reversible. *Transl. Psychiatry* 2 e131. 10.1038/tp.2012.59 22760555 PMC3410630

[B81] Stewart M. G. Davies H. A. Sandi C. Kraev I. V. Rogachevsky V. V. Peddie C. J. (2005). Stress suppresses and learning induces plasticity in CA3 of rat hippocampus: a three-dimensional ultrastructural study of thorny excrescences and their postsynaptic densities. *Neuroscience* 131 43–54. 10.1016/j.neuroscience.2004.10.031 15680690

[B82] Thompson S. M. Kallarackal A. J. Kvarta M. D. Van Dyke A. M. LeGates T. A. Cai X. (2015). An excitatory synapse hypothesis of depression. *Trends Neurosci.* 38 279–294. 10.1016/j.tins.2015.03.003 25887240 PMC4417609

[B83] Timmermans W. Xiong H. Hoogenraad C. C. Krugers H. J. (2013). Stress and excitatory synapses: from health to disease. *Neuroscience* 248 626–636. 10.1016/j.neuroscience.2013.05.043 23727506

[B84] Veeraiah P. Noronha J. M. Maitra S. Bagga P. Khandelwal N. Chakravarty S. (2014). Dysfunctional glutamatergic and γ-aminobutyric acidergic activities in prefrontal cortex of mice in social defeat model of depression. *Biol. Psychiatry* 76 231–238. 10.1016/j.biopsych.2013.09.024 24239130

[B85] Wang M. Perova Z. Arenkiel B. R. Li B. (2014). Synaptic modifications in the medial prefrontal cortex in susceptibility and resilience to stress. *J. Neurosci.* 34 7485–7492. 10.1523/JNEUROSCI.5294-13.2014 24872553 PMC4035514

[B86] Wiborg O. (2013). Chronic mild stress for modeling anhedonia. *Cell Tissue Res.* 354 155–169. 10.1007/s00441-013-1664-0 23801433

[B87] Wilber A. A. Walker A. G. Southwood C. J. Farrell M. R. Lin G. L. Rebec G. V. (2011). Chronic stress alters neural activity in medial prefrontal cortex during retrieval of extinction. *Neuroscience* 174 115–131. 10.1016/j.neuroscience.2010.10.070 21044660 PMC3020264

[B88] Willner P. (1997). Validity, reliability and utility of the chronic mild stress model of depression: a 10-year review and evaluation. *Psychopharmacology (Berl.)* 134 319–329. 10.1007/s002130050456 9452163

[B89] Willner P. (2005). Chronic mild stress (CMS) revisited: consistency and behavioural-neurobiological concordance in the effects of CMS. *Neuropsychobiology* 52 90–110. 10.1159/000087097 16037678

[B90] Willner P. (2016a). Reliability of the chronic mild stress model of depression: a user survey. *Neurobiol. Stress* 6 68–77. 10.1016/j.ynstr.2016.08.001 28229110 PMC5314419

[B91] Willner P. (2016b). The chronic mild stress (CMS) model of depression: history, evaluation and usage. *Neurobiol. Stress* 6 78–93. 10.1016/j.ynstr.2016.08.002 28229111 PMC5314424

[B92] Willner P. Muscat R. Papp M. (1992). Chronic mild stress-induced anhedonia: a realistic animal model of depression. *Neurosci. Biobehav. Rev.* 16 525–534. 10.1016/S0149-7634(05)80194-0 1480349

[B93] Xiao Q. Wang F. Luo Y. Chen L. Chao F. Tan C. (2018). Exercise protects myelinated fibers of white matter in a rat model of depression. *J. Comp. Neurol.* 526 537–549. 10.1002/cne.24350 29098693

[B94] Yuen E. Y. Wei J. Liu W. Zhong P. Li X. Yan Z. (2012). Repeated stress causes cognitive impairment by suppressing glutamate receptor expression and function in prefrontal cortex. *Neuron* 73 962–977. 10.1016/j.neuron.2011.12.033 22405206 PMC3302010

